# Taxon abundance, diversity, co-occurrence and network analysis of the ruminal microbiota in response to dietary changes in dairy cows

**DOI:** 10.1371/journal.pone.0180260

**Published:** 2017-07-13

**Authors:** Ilma Tapio, Daniel Fischer, Lucia Blasco, Miika Tapio, R. John Wallace, Ali R. Bayat, Laura Ventto, Minna Kahala, Enyew Negussie, Kevin J. Shingfield, Johanna Vilkki

**Affiliations:** 1 Green Technology, Natural Resources Institute Finland, Jokioinen, Finland; 2 Bio-based business and industry, Natural Resources Institute Finland, Jokioinen, Finland; 3 Rowett Institute of Nutrition and Health, University of Aberdeen, Aberdeen, United Kingdom; 4 Institute of Biological, Environmental and Rural Sciences, Aberystwyth University, Aberystwyth, United Kingdom; Wageningen University, NETHERLANDS

## Abstract

The ruminal microbiome, comprising large numbers of bacteria, ciliate protozoa, archaea and fungi, responds to diet and dietary additives in a complex way. The aim of this study was to investigate the benefits of increasing the depth of the community analysis in describing and explaining responses to dietary changes. Quantitative PCR, ssu rRNA amplicon based taxa composition, diversity and co-occurrence network analyses were applied to ruminal digesta samples obtained from four multiparous Nordic Red dairy cows fitted with rumen cannulae. The cows received diets with forage:concentrate ratio either 35:65 (diet H) or 65:35 (L), supplemented or not with sunflower oil (SO) (0 or 50 g/kg diet dry matter), supplied in a 4 × 4 Latin square design with a 2 × 2 factorial arrangement of treatments and four 35-day periods. Digesta samples were collected on days 22 and 24 and combined. QPCR provided a broad picture in which a large fall in the abundance of fungi was seen with SO in the H but not the L diet. Amplicon sequencing showed higher community diversity indices in L as compared to H diets and revealed diet specific taxa abundance changes, highlighting large differences in protozoal and fungal composition. *Methanobrevibacter ruminantium* and *Mbb*. *gottschalkii* dominated archaeal communities, and their abundance correlated negatively with each other. Co-occurrence network analysis provided evidence that no microbial domain played a more central role in network formation, that some minor-abundance taxa were at nodes of highest centrality, and that microbial interactions were diet specific. Networks added new dimensions to our understanding of the diet effect on rumen microbial community interactions.

## Introduction

The rumen contains a highly complex microbial ecosystem comprising several thousand species of bacteria [1], of which 30 groups are dominant [2], 12 genera of ciliate protozoa [2], 6 genera of anaerobic fungi [3], and approximately 10 main taxa of methanogenic archaea [4], as well as variable abundances of mycoplasmas, bacteriophages, archaeophages and viruses that infect the eukaryotic microorganisms. Describing the community and especially its relationship to function is challenging, the latter partly because of the redundancy of function across different taxa [2,5]. Taxon abundances and diversity based on ssu rRNA amplicon analyses are used to evaluate microbial community responses to experimental treatments, time, health or developmental stage of an animal. These data assessed in relation to animal phenotype often lead to retrospective attempts to explain differences in function, such as methanogenesis or feed efficiency [6–8]. However, most of the analytical techniques concentrate on highlighting properties of individual species, leaving the interactions within the microbial community unexplored. Microbial co-occurrence, estimated as positive correlation between taxa, indicates to some extent similarity of function or similarity in response to the same environmental conditions, but to our knowledge few studies have applied such analysis in the rumen [9,10]. Potential interactions between microbial taxa can be investigated further by network analysis of significant taxa co-occurrence patterns, obtained from sequence data. Network analysis approaches have been successfully used in other ecosystems, including marine [11], soil [12] or the human gut [13]. It expands upon information generated using standard diversity analysis tools and offers an approach to identify patterns of microbial interactions in ecosystems occupied by uncultured microorganisms.

Rumen microbial community structure is affected by ruminant species [9], breed [14], or age of animals [10]. However, diet is the major determinant of ruminal microbial composition [2]. Diet depends on the geographical location and the type of production system. While feedlot cattle are often fed diets based on grain and rich in fermentable carbohydrates, the Nordic dairy sector depends on grass forage based diets, fed mainly in the form of restrictively fermented silage [15]. Enrichment of diets with plant oils or oilseeds has been explored as a way to increase dietary energy supply in dairy cows and alter fatty acid composition in milk and meat [16]. Further, lipid supplements are known to reduce enteric methane production [17], therefore providing options to lower the environmental impact of animal production.

The aim of the present study was to explore the value of stepwise increases in the depth of community analytical tools in understanding responses to two commercially important dietary changes in dairy cows, namely transition from forage to concentrate diets and the supplementation of diets with vegetable oil. The results indicate that network analysis based on ssu RNA amplicon sequencing adds new dimensions to our understanding of the rumen microbial community.

## Materials and methods

### Animals, experimental design and diets

The animal study research protocol was approved by the National Ethics Committee (ESAVI/794/04.10.03/2011, Kuopio, Finland) in accordance with the guidelines established by the European Community Council Directives 86/609/EEC [18]. Details on the experimental design have been reported by [19]. In summary, four multiparous Nordic Red dairy cows fitted with rumen cannulae in mid lactation were used in a 4 × 4 Latin Square experiment with a 2 × 2 factorial arrangement of treatments and four 35 day experimental periods. Treatments consisted of iso-nitrogenous total mixed ration based on grass silage containing a low (65:35) or high (35:65) proportion of concentrates supplemented with 0 (L and H, respectively) or 50 g/kg diet DM of sunflower oil (SO) (LSO and HSO, respectively). The forage was restrictively fermented grass silage prepared from mixed timothy meadow fescue swards treated with a formic acid based ensiling additive. Concentrates comprised variable amounts of ground wheat, rolled barley, rapeseed expeller, urea and mineral and vitamin pre-mix [19]. Experimental diets were fed for 26 days followed by 9 days on L diet to minimize treatment carry-over effects.

### Rumen sample collection and DNA extraction

Ruminal digesta was sampled on day 22 at 0900 and on day 24 at 1500 of each experimental period. Representative samples were collected from four sites within the reticulorumen (anterior dorsal, anterior ventral, posterior dorsal, and posterior ventral) and composited by manual mixing. Twenty five grams of digesta collected at each time point was preserved in 50 mL of phosphate buffered saline-glycerol (30% v/v), frozen and stored at -80°C until DNA extraction. Total genomic DNA was extracted from combined d 22 and d 24 sub-samples following the protocol of Yu and Morrison [20].

### Quantitative real-time PCR analysis

The total amounts of bacteria, archaea, ciliate protozoa and fungi were measured by qPCR. Primers used for amplification are described in S1 Table. qPCRs were performed in volumes of 12.5 μl using 10 ng of template DNA (50 ng for ciliates), forward and reverse primers (400 nM) and 2x qPCRBIO SyGreen Mix Lo-ROX (PCR Biosystems). Amplification conditions were initial denaturation at 95°C for 5 min, followed by 60 cycles at 95°C for 15 s, annealing temperatures specific for each locus (as indicated in S1 Table) for 30 s, 72°C for 30 s, and 95°C for 15 s. PCRs were carried out in triplicate, including negative controls, on a LightCycler® 480 System (Roche, Mannheim, Germany). The absolute quantity of test samples was estimated against serial dilutions of DNA standards, amplified to generate calibration curves. Methods used to generate standards for bacteria, archaea, ciliate protozoa and fungi are described in detail in [21,22]. The quality of generated calibration curves was R^2^ = 0.99 for bacteria and fungi and R^2^ = 0.98 for archaea and ciliate protozoa, respectively. Microbial abundances were calculated from triplicate Ct values using the universal calibration equation. Differences between diets were tested for significance using a paired *t*-test.

### Amplicon sequencing

Rumen microbial diversity was assessed by amplicon sequencing of bacteria using a 454 GS Junior (Roche) at Luke (formerly known as MTT Agrifood Research Finland). Archaea, ciliate protozoa and fungi diversity were evaluated using MiSeq (Illumina) platforms. The change in sequencing platform was related to the rapid development of Illumina sequencing capacity at the time when sequencing was done and lower costs in relation to Roche 454. Primer sequences used for amplification are described in S1 Table. For bacteria, five 10 bp barcodes (Roche TCB No. 005–2009) were added at the 5’-end of both forward and reverse primers to make amplicon pooling possible. The PCR amplification, library preparation and sequencing on a 454 GS Junior (Roche) were performed following standard procedures (S1 Text). Sequencing of archaeal libraries was performed at Fasteris SA (Geneva, Switzerland) as described in [23]. Ciliate protozoa and anaerobic fungi libraries were constructed and sequenced at LGC Genomics (Berlin, Germany) following the 250 bp paired-end protocol.

### Sequence data processing

Bacterial sequences were demultiplexed based on adaptor and primer barcodes using a custom script written in python. Reads shorter than 200nt in length and with a minimum quality score lower than 25 were discarded. No mismatches in primer sequence or ambiguous bases were allowed. Archaeal sequences were processed as described in [23]. Demultiplexing, adapter removal and merging of paired-end reads of protozoal and fungal sequences were done by sequencing data provider LGC Genomics (Berlin, Germany) following standard in-house procedures. Sequences were clustered into operational taxonomic units (OTU) at 97% similarity using Uclust [24] and filtered from chimeric reads using ChimeraSlayer or UCHIME (for anaerobic fungi) as implemented in Qiime v1.7.0 [25]. Taxonomy was assigned using BLAST [26], comparing bacterial 16S rRNA sequences against Greengenes 12_10 [27], archaeal 16S rRNA sequences to RIM-DB [28], protozoal 18S rRNA sequences to SILVA 18S [29] and anaerobic fungi using a curated fungal ITS reference database [30]. Singleton OTUs were removed prior to further analyses resulting in 4,902,197 non-chimeric high quality reads in total (S1 Text).

### Statistical analyses

From taxonomic sequence information, microbial community alpha diversity was estimated using Simpson’s index of diversity (1-D). Species richness and Pielou’s measure of species evenness were calculated using R package *vegan* [31]. Beta diversity or grouping of samples based on diet was evaluated by using Bray-Curtis as dissimilarity measure and nonmetric multidimensional scaling (NMDS) as ordination method. Distance-based permutational multivariate analysis of variance (adonis) was used to assess significant differences with respect to diet. Significance was defined at *P* < 0.05 after 999 permutations as implemented in *vegan*. The impact of diet on individual rumen microbial taxa was analyzed using the Mixed procedure of SAS (v 9.2, SAS institute, Cary, NC) including fixed effects of period, forage to concentrate ratio (FC), SO, and FC by SO interactions, and random effect of cow. Microbial counts were normalized with a log_2_ transformation and centered so that abundance profiles for all samples exhibited a mean of 0 and a standard deviation of 1. Data normality was checked using the Shapiro-Wilk test. Statistically significant differences were declared at P ≤ 0.05. A *post-hoc* pairwise comparison of all diets was performed using Tukey’s test.

Microbial networks were created using the merged data for three different combinations of diet changes: (i) from high to low concentrate diets (H-L), (ii) low concentrate diets with and without SO supplement (L-LSO), and (iii) high concentrate diets with and without SO supplement (H-HSO). From the 126 taxa at the genus level, those with less than 2 reads per sample across all diets were first filtered out, resulting in a total of 76 remaining taxa. The absolute counts were log-transformed and for each combination (i)-(iii) the partial correlations were calculated using pairwise complete observations, therefore excluding the fungal population from H-HSO comparison. Partial correlations were calculated by *EBICglasso* in *qgraph* -package [32] based on the nearest positive definite correlation matrix computed using *nearPD* -function in *Matrix* package [33]. *EBICglasso* selects the tuning parameter using the Extended Bayesian Information criterion and computes a sparse Gaussian graphical model with the graphical lasso [34]. The *qgraph* package was also used for drawing the partial correlation networks presenting the direct biological interactions. Betweennes (shortest distance between all pairs of nodes through the focal node) was calculated using the absolute values of (partial) correlation as the edge weight. Difference in mean betweenness among the microbial groups (bacteria, archaea, ciliate protozoa, fungi) was tested with One Way Anova. Binomial test for vicariance was performed using function *binom*.*test* in R [35]. As every edge is connected to only two nodes, by definition, the pattern of edge/node connections has a binomial expectation [36].

## Results

### qPCR analysis

**Fig 1:**
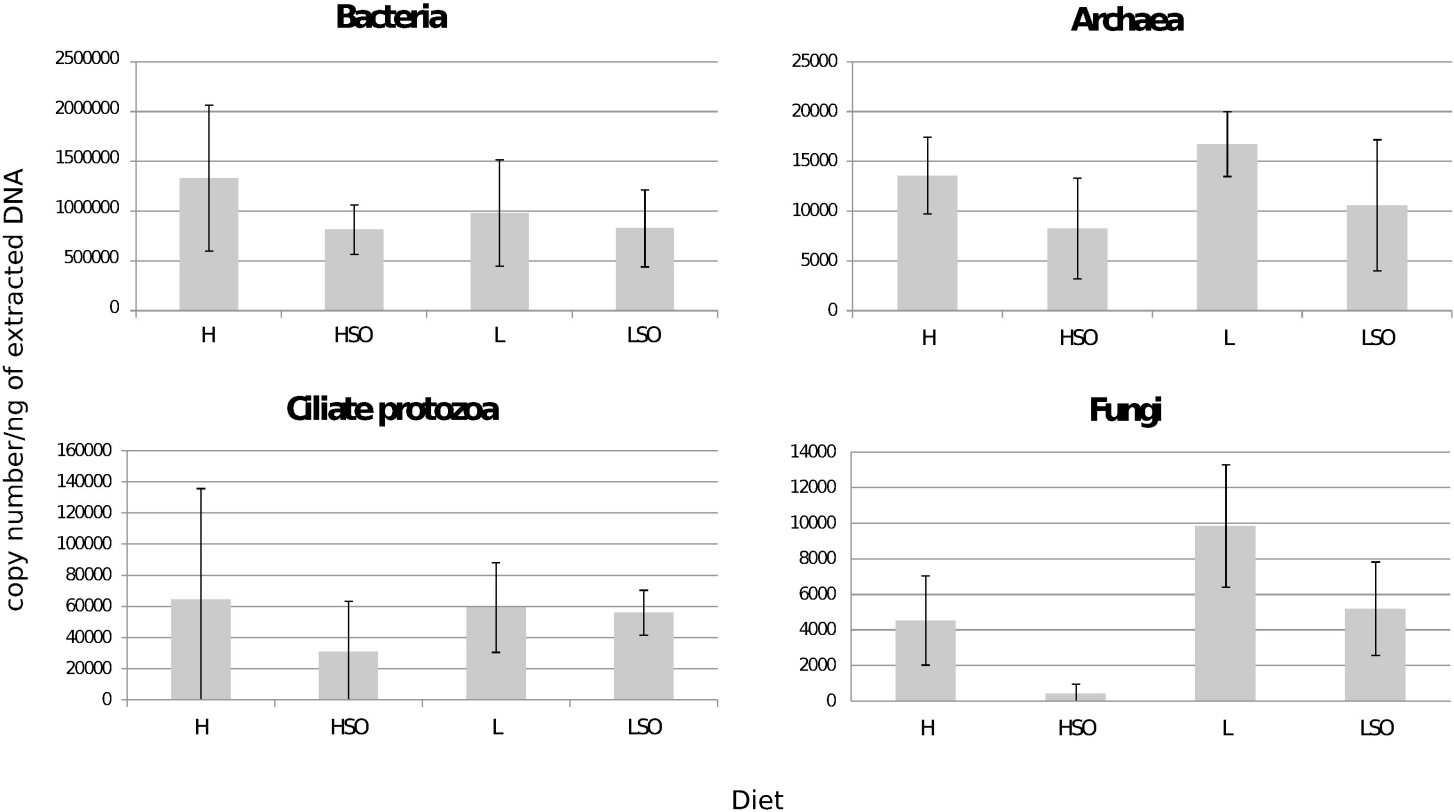
qPCR of bacterial 16S rRNA, archaeal *mcr*A, protozoal 18S rRNA genes and fungal region between 18S rRNA and ITS1 of rumen digesta samples. Samples collected from lactating cows fed four diets: high (H) or low (L) proportion of concentrates without oil, or supplemented with SO (HSO and LSO, respectively). Results are expressed as copy number per ng of extracted DNA. Error bars represent standard deviations (SD), n = 4 per diet.

Real-time qPCR targeting bacterial 16S rRNA, archaeal *mcr*A, ciliate protozoa 18S rRNA genes and for fungi targeting the region between 18S rRNA and ITS1 were used to quantify the abundance changes of ruminal microorganisms across the four diets. SO when supplemented to both H and L diets decreased total amounts of all rumen microbial groups: bacteria, archaea, ciliate protozoa and fungi (Fig 1). However, due to high between-sample variability, differences were significant for fungi only. Fungi were two times more abundant in L as compared to the H treatment (*P* = 0.03). SO significantly decreased the total abundance of fungi in both HSO and LSO diets but the effect was strongest in HSO (H vs HSO by 10.5-fold, *P* = 0.03; L vs LSO by 1.9-fold, *P* = 0.04; Fig 1).

### Abundance and diversity derived from amplicon sequencing

**Fig 2:**
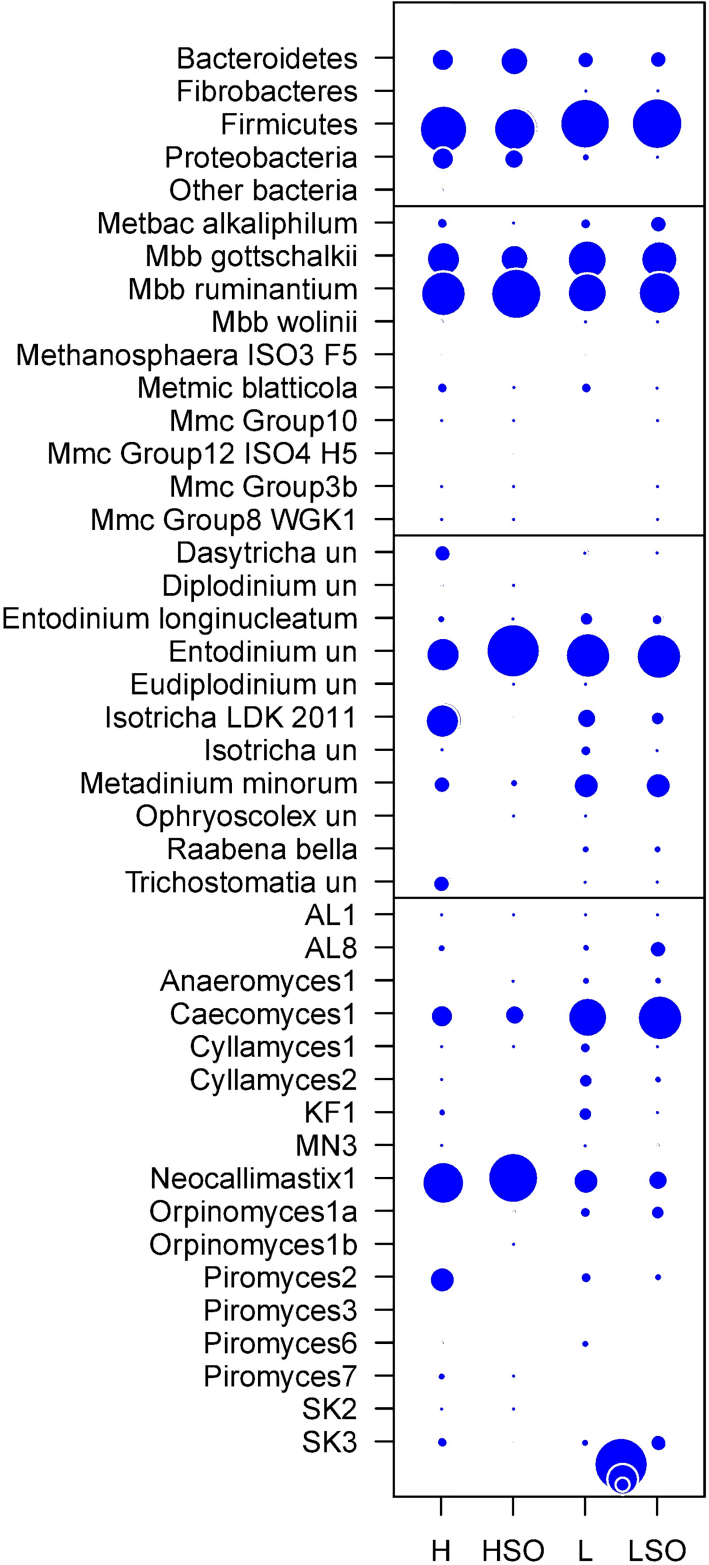
Relative abundance of bacteria, archaea, ciliate protozoa and fungi among diets. Bubble-charts represent taxonomy at the phylum level for bacteria and genus level for the rest. Diets are as follows: high (H) or low (L) proportion of concentrates without oil, or supplemented with SO (HSO and LSO, respectively).

Amplicon sequencing showed that rumen bacterial community was dominated by Firmicutes (average per diet from total 55.9–86.8%), Bacteroidetes (8–24.4%) and Proteobacteria (0.9–13.4%). Abundance of minor bacterial phyla (Actinobacteria, Cyanobacteria, Spirochaetes, Synergisteles, TM7 and Tenericutes) was below 0.5% (Fig 2). *Lachnospiraceae*, *Ruminococcaceae*, *Clostridiaceae*, *Veillonellaceae* and *Catabacteriaceae* families were the most abundant among Firmicutes, while Bacteroidetes were mainly represented by *Prevotellaceae*, and *Succinivibrionaceae* was the most abundant family among Proteobacteria (S2 Table).

The archaeal community was dominated by *Methanobrevibacter ruminantium* (46–74%) and *Mbb*. *gottschalkii* (24–46%), with *Methanobacterium alkaliphilum*, *Methanimicrococcus blatticola*, *Mbb*. *wolinii*, *Methanosphaera ISO3-F5* and several groups from the *Methanomassiliicoccaceae* family observed at lower abundance (Fig 2).

The most numerous protozoa were *Entodinium* (33.5–96.2%), *Isotricha* LDK 2011 (0.3–36.3%), *Metadinium minorum* (1.7–19.7%), *Dasytricha* (0.1–7.3%) and *Entodinium longinucleatum* (1.3–4.8%).

The fungal community was represented by six well characterized and six previously identified novel fungal groups (AL1, AL8, KF1, MN3, SK2 and SK3). *Neocallimastix* 1 (11.8–84.5%) and *Caecomyces* 1 (10.3–57.5%) were the predominant genera in all diets. FC ratio and SO strongly influenced the abundance of *Cyllamyces* 1 and 2, *Orpinomyces* 1a, and *Piromyces* 6 and 7, and novel groups AL8, KF1, SK3 (Fig 2).

**Fig 3:**
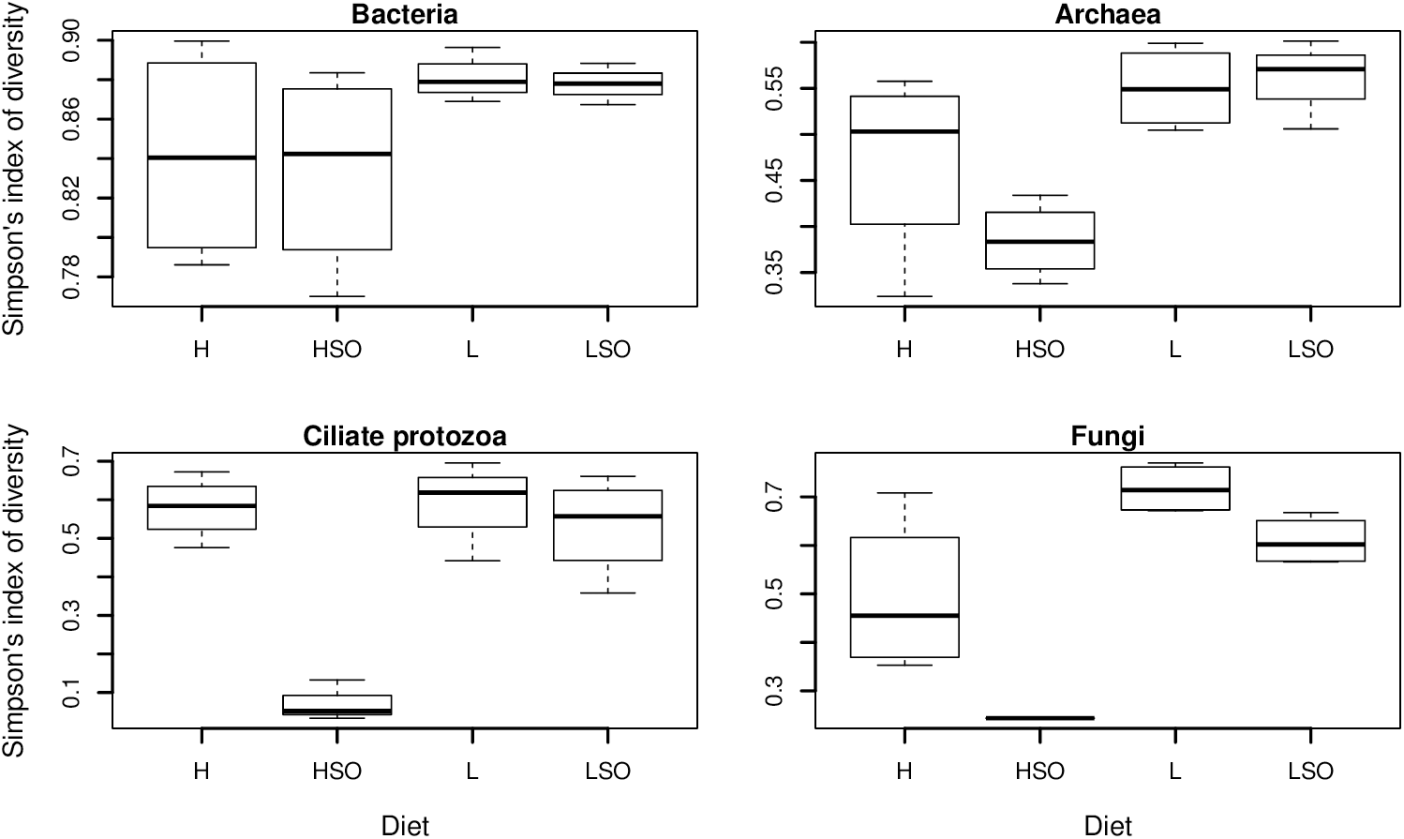
Bacterial, archaeal, protozoal and fungal diversity (Simpson’s index) as measured from amplicon sequence data in four diets. Diets are as follows: high (H) or low (L) proportion of concentrates without oil, or supplemented with SO (HSO and LSO, respectively).

Simpson’s index of diversity was greater in L diets and lowest in HSO (Fig 3). Higher diversity in the L and LSO treatments was related to higher microbiota richness (S1 Fig) as well as higher evenness (S2 Fig).

**Fig 4:**
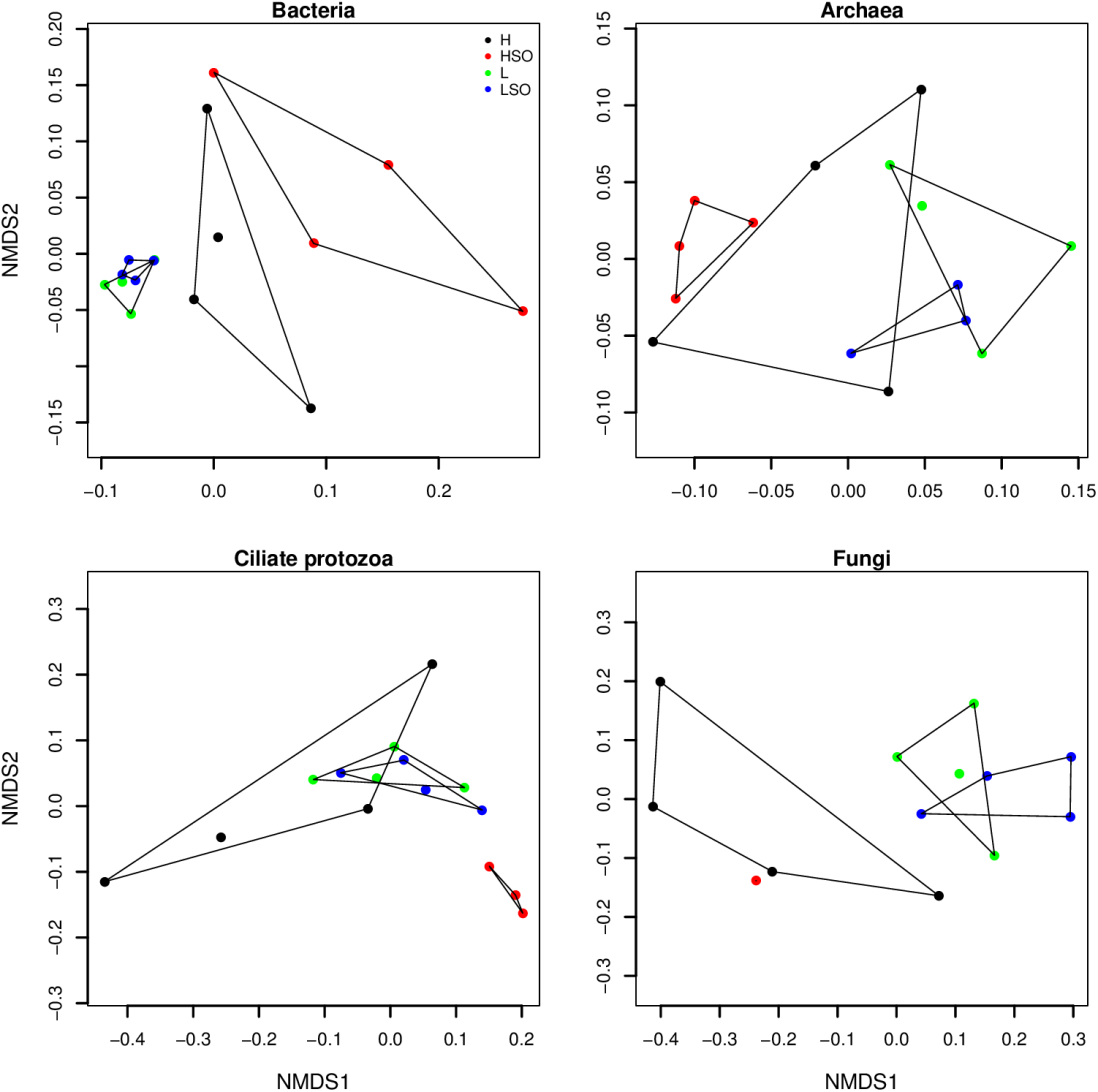
NMDS plots of bacteria, archaea, ciliate protozoa and fungi, based on amplicon sequence data. Individual animals are represented by dots, colored based on the diet: high (H—black) or low (L—green) proportion of concentrates without oil, or supplemented with SO (HSO–red, and LSO—blue).

The structure of overall rumen microbial populations, assessed by two-dimensional NMDS plots based on Bray-Curtis dissimilarities, showed marked differences between diets in bacteria, archaea, ciliate protozoa and fungi, respectively (adonis analysis for diets *P* < 0.05) (Fig 4). The variance between the animals within the same diet was higher in H-HSO compared to L-LSO treatments. The FC ratio alone significantly affected bacterial, archaeal and fungal (adonis analysis *P* < 0.05) but not ciliate community structure.

### Dietary effects on individual microbial taxa

The effects of diet were evaluated on 37 genus-like taxonomic groups of bacteria, 11 groups of archaea, 14 of ciliate protozoa and 16 of fungi, respectively. FC ratio significantly changed the abundance of 46% of bacterial, 30% of archaeal and 21% of ciliate protozoa groups. The effect of SO supplementation was less pronounced (Table 1).

**Table 1:** Changes in rumen microbial community abundances from amplicon sequence data in four diets, represented as average abundance per diet ± standard deviation (SD). Diets are as follows: high (H) or low (L) proportion of concentrates without oil, or supplemented with SO (HSO and LSO, respectively). Only taxa significantly affected by diet are presented. The small letter at the beginning of each bacterial taxon name identifies the deepest classification level to which the OTUs were assigned. Diet effect represents significant (*P* < 0.05) microbial abundance changes due to FC, SO or FC × SO interaction.

	Abundance (%) ± SD				
Taxon	H	HSO	L	LSO	Diet effect
p_Bacteroidetes un	0.22±0.26	0.14±0.09	0.72±0.73	0.45±0.47	FC
o_Bacteroidales un1	0.13±0.06	0.81±0.47	0.20±0.13	0.20±0.10	SO
f_Prevotellaceae un1	0.33±0.18	0.82±0.37	0.28±0.19	0.38±0.19	FC,SO
f_Prevotellaceae un2	0	0.01±0.01	0.05±0.01	0.01±0.03	FC×SO
g_Prevotella	12.69±6.55	24.01±10.9	7.76±4.05	6.99±1.57	FC
g_Fibrobacter	0.44±0.38	0.01±0.03	0.68±0.25	0.55±0.20	FC,SO,FC×SO
c_Clostridia un1	7.28±1.03	4.79±1.14	16.03±1.72	14.01±0.96	FC
o_Clostridiales un1	2.55±0.78	2.54±1.02	3.69±0.91	4.34±1.13	FC
o_Clostridiales un2	0.70±0.18	0.30±0.20	0.91±0.18	0.61±0.08	SO
f_Clostridiaceae un2	1.17±0.53	0.56±0.43	1.91±0.23	2.07±0.33	FC
g_Clostridium	0.59±0.26	0.24±0.20	0.80±0.08	0.76±0.22	FC
f_Lachnospiraceae un2	2.30±0.86	1.60±0.96	4.47±1.09	3.98±0.37	FC
g_Anaerostipes	0.11±0.10	0.09±0.06	0.43±0.19	0.44±0.18	FC
g_Coprococcus	1.65±0.31	1.57±1.23	2.68±0.38	3.10±0.93	FC
g_Shuttleworthia	0.64±0.41	0.28±0.20	0.34±0.10	0.19±0.09	FC,SO
g_[Ruminococcus]	0.14±0.10	0.01±0.02	0.11±0.04	0.14±0.05	FC×SO
f_Ruminococcaceae un1	2.22±1.18	2.99±0.69	3.24±0.65	3.77±0.51	SO
f_Ruminococcaceae un2	0.28±0.14	0.08±0.06	0.29±0.11	0.76±0.42	FC×SO
g_Succiniclasticum	0.29±0.17	0.27±0.18	0.12±0.07	0.09±0.02	FC
o_Coriobacteriales un	1.51±0.53	1.18±1.14	3.51±0.58	3.51±0.55	FC
c_Gammaproteobacteria un	0.12±0.10	0.73±1.21	0.05±0.07	0.03±0.02	FC
f_Succinivibrionaceae un	0.05±0.06	0.11±0.12	0.01±0.03	0.02±0.03	FC
f_Succinivibrionaceae un2	3.95±3.21	4.80±5.05	0.49±0.41	0.78±0.70	FC
Methanobacterium alkaliphilum	1.87±3.64	0	2.95±3.56	7.55±3.52	FC,FC×SO
Methanobrevibacter ruminantium	61.84±14.5	74.48±2.82	48.08±6.43	53.83±9.03	FC
Methanobrevibacter wolinii	0.5±0.81	0.14±0.30	0	0.01±0.01	FC
Methanosphaera ISO3 F5	0.25±0.11	0.35±0.10	0.33±0.08	0.46±0.08	SO
Methanomassiliicoccaceae Group10	0.01±0.01	0	0.1±0.1	0	FC×SO
Methanomassiliicoccaceae Group3b	0.01±0.01	0.03±0.04	0.06±0.07	0.01±0.01	FC×SO
Entodinium uncultured	33.55±21.5	96.21±2.81	57.24±12.7	63.45±11.80	FC,SO,FC×SO
Isotricha LDK 2011	36.28±31.7	0.26±0.07	10.06±11.2	6.24±8.54	SO
Metadinium minorum	8.79±7.64	1.74±1.58	19.70±5.35	19.68±4.67	FC
Polydiniella mysorea	0	0	0.01±0	0	FC×SO
Raabena bella	0.11±0.09	0.06±0.02	1.97±0.85	2.07±1.87	FC
Caecomyces1	16.38±17.09	10.3	44.34±9.68	57.52±4.24	SO
KF1	1.53±2.54	0.17	6.67±7.29	0.85±1.23	SO

The bacteria whose abundance was significantly affected by diet belonged to Firmicutes, Bacteroidetes, Proteobacteria and Fibrobacteres phyla. *Clostridium*, *Anaerostipes* and *Coprococcus* genera were more abundant in L diets, while *Shuttleworthia* (family *Lachnospiraceae*) and *Succiniclasticum* (family *Veillonellaceae*) as well as two genus-like groups within *Succinivibrionaceae* family were more frequent when H diets were fed. Among Bacteroidetes, *Prevotella* was detected at three times higher abundance in association with HSO (24%) compared to L diets (7.0–7.8%). The HSO diet significantly decreased the abundance of *Fibrobacter*. However, diet did not significantly affect abundance of *Mogibacterium*, *Butyrivibrio*, *Moryella*, *Pseudobutyrivibrio*, *Oscillospira*, *Ruminococcus* or *Ruminobacter*.

Among archaea, *Mbb*. *ruminantium* was more abundant in H, while *Mbb*. *gottschalkii* was predominant in L diets. *Methanobacterium alkaliphilum* was most abundant in LSO (7.6%) but absent from HSO diet, while *Mbb*. *wolinii* was more abundant in HSO-H treatments (0.1–0.5%) compared to L diets. Although *Methanosphaera ISO3-F5* was observed in all four diets at low abundance, it favored treatments with SO supplement more than without. In contrast, the *Methanomassiliicoccaceae* Group 10 was present at low abundance only in diets without SO.

The FC ratio and SO had varying effects on rumen ciliate protozoa. *Entodinium* dominated in all diets but it exhibited lowest abundance in H (33.5%) and highest in HSO (96.2%) diet. L treatments positively affected the abundance of *Metadinium minorum* and *Raabena bella*, while *Isotricha* LDK-2011 was sensitive to SO and was at highest abundance in H (36.3%) and almost absent in HSO (0.3%) treatments (Table 1).

Anaerobic fungal diversity was strongly influenced by diet, however, due to high between-animal variation within the same diet, statistically significant changes were detected only in *Caecomyces* 1 and KF1 (Table 1). *Caecomyces* 1 was dominant in L-LSO and *Neocallimastix* 1 in H-HSO diets. Further, L had higher proportion of KF1 (6.7%), *Cyllamyces* 2 and 1 (6% and 4%) and *Piromyces* 6 (2.4%), but *Piromyces 2* (19.8%) and *Piromyces* 7 (2.1%) were more abundant in H (Fig 2).

### Network analysis

Many of the metabolic functions of ruminal microorganisms occur across domains and phyla, but often microorganisms share less common properties that link them into groups in which members are species from different high-level groupings. For example, primary degraders provide nutrients for a secondary community, then for tertiary degraders and so on. It might be predicted that both interdependence and competition between taxa should be apparent from community analysis, particularly in their responses to dietary changes. Various depths of network analysis were therefore undertaken to investigate these interdependencies.

Bacteria are the most abundant and diverse group of ruminal microorganisms. Therefore we tested if bacteria play a more central role in network formation compared to protozoa, fungi and archaea. The assessment was based on betweenness centrality values for each microorganism and tested for group effect on centrality with one way ANOVA. Betweenness centrality is an indicator of taxon centrality in the network. It is equal to the number of shortest paths from all vertices to all others that pass through that node (taxon). A node with high betweenness centrality has a large influence on the interaction through the network, under the assumption that interaction follows the shortest paths. In all dietary comparisons (L-H, L-LSO, H-HSO) the absence of significant results (*P* > 0.3) suggested that bacteria alone do not fulfil a central role in network formation.

**Fig 5:**
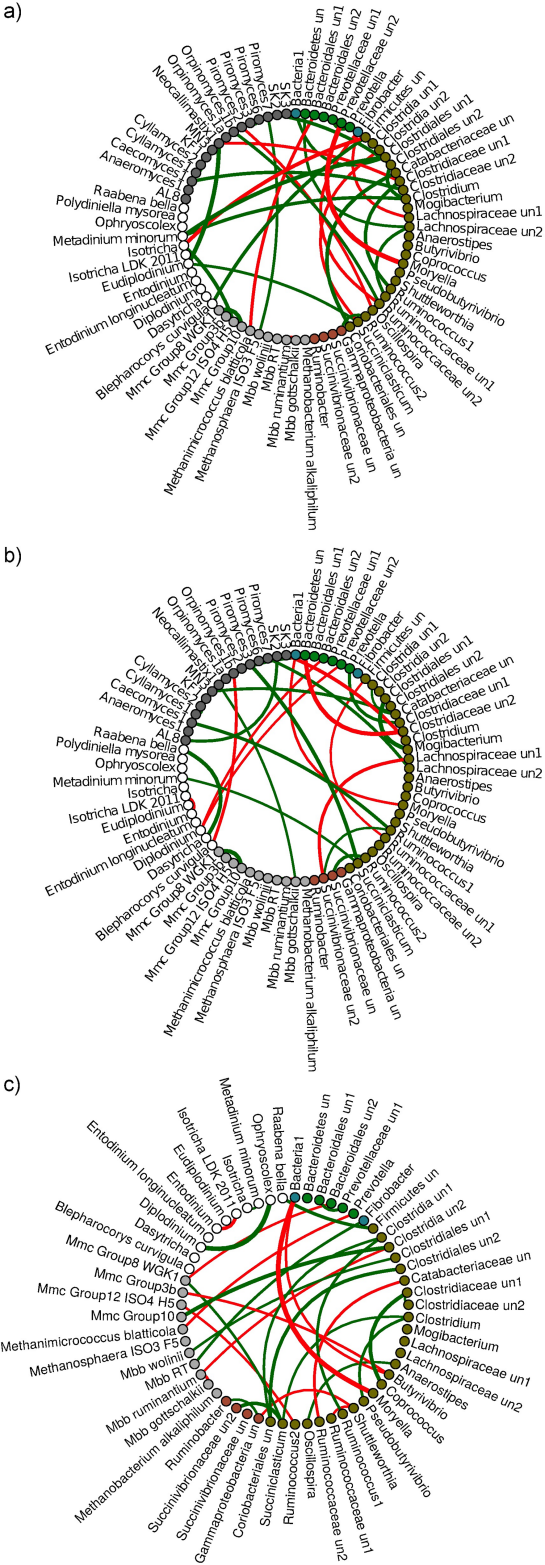
Graphical networks representing the interactions between rumen microorganisms within three diet comparisons. a) L-H, b) L-LSO, and c) H-HSO diet comparisons. Nodes correspond to microbial taxa while green and red edges represent positive and negative partial correlations above 0.25, respectively. Microbial taxa are colored by taxonomy: archaea—light gray, ciliate protozoa—white, fungi—dark grey, bacteria: Bacteroidetes—green, Firmicutes—moss green, Proteobacteria—brown, others—blue.

We further tested whether bacteria interact more within their own domain as compared to interactions with ciliate protozoa, fungi or archaea. Here we analysed networks at two weight levels, first when including links with partial correlation > 0.05, secondly with only links above 0.25 partial correlation. By limiting to a correlation threshold of > 0.25, few and only the most robust interactions between the taxa were evaluated (Fig 5). Most of ruminal microorganisms create networks fulfilling the first, less stringent criterion (S3–S5 Figs). By analyzing the data at the > 0.05 level, the resulting networks did not show that bacteria would interact more often with other bacteria as compared to other microbial groups in L-LSO and L-H (vicariance test *P* > 0.05), but did in H-HSO (*P* = 0.015). When only the most robust interactions were taken into account (>0.25), between-bacteria interactions were significantly more abundant than compared between bacteria and other groups in L-LSO (*P* = 0.026) and were close to significance in L-H (*P* = 0.065) and H-HSO (*P* = 0.077). This suggests that at least strong direct interaction is more common within bacteria domain.

Following these observations we investigated microbial taxa that act as main information gateways (hubs) in networks. For this we analysed betweenness centrality data and identified the microorganisms with highest centrality values (>1.5). A node with high betweenness centrality could mean that the microorganism plays a central part in the flow of information from one part of the network to the other. Microorganisms with the most enhanced centrality in the L-H comparison were Clostridiales un2, *Clostridium*, *Shuttleworthia*, *Succiniclasticum*, *Eudiplodinium*, *Metadinium minorum*, *Neocallimastix*1 and *Piromyces*3. In L-LSO betweenness was highest in Bacteroidales un1, Bacteroidales un2, *Anaerostipes*, *Ruminococcus*1, Ruminococcaceae un1, Succinivibrionaceae un2, *Orpinomyces*1a and *Orpinomyces*1b. In H-HSO, the highest centrality values were observed for Bacteroidales un2, *Succiniclasticum* and *Methanomassiliicoccaceae* Group 8 WGK1.

We further elucidated if microbial taxa significantly affected by dietary changes appear in the networks. For this we screened networks generated with the more stringent filtering parameter (partial correlation > 0.25) as to whether diet affected taxa (Table 1) belong to the most robust correlations or not. In the L-H case, Lachnospiraceae un2, whose abundance decreased significantly from the L to H diet, was detected in strong positive correlation with Ruminococcaceae un1 and Bacteroidetes un (Fig 5A). Other microbes significantly affected by diet, like Succinivibrionaceae un2, *Anaerostipes*, *Clostridium* un1 and *Raabena bella*, were not detected in the most robust partial correlations. In the L-LSO comparison, no bacteria or ciliate protozoa were significantly affected by dietary change. Only the *Methanomassiliicoccaceae* Group 10 from archaea significantly decreased while fungi AL8 and SK2 significantly increased in LSO (S2 Table). All three were detected in robust associations (Fig 5B). In H-HSO, microbial taxa that were significantly affected by dietary switch were present within the strongest partial correlations as well. The abundance of Ruminococcus1 from the *Lachnospiraceae* family significantly decreased in HSO (S2 Table) and was observed in a positive correlation within a network of Clostridium un1—Coriobacteriales un—Clostridiales un2 (Fig 5C). Prevotellaceae un1 and Bacteroidales un1 abundance significantly increased in HSO and both were positively correlated, while Ruminococcaceae un1 was negatively associated with *Shuttleworthia*. *Methanomassiliicoccaceae* Group 3b, although at low abundance, was negatively associated with *Butyrivibrio*. *Entodinium* and *Isotricha* LDK 2011 were in a negative correlation. It was concluded that taxa significantly affected by diet do not appear in isolation but co-occur in microbial networks with other taxa, which may indicate their metabolic interaction during diet changes.

We further studied if microbial taxa, detected in the most robust between- and within—domain co-occurrences, are the same irrespective of diet. For that we explored the networks of the most robust interactions (> 0.25 partial correlation) for each diet change separately and show that they are diet specific (Fig 5). In the L-H comparison, the density of connections within and between domains indicated that bacteria were slightly more densely connected with other bacteria (1.8%) as compared with density in connections within archaeal, fungal or protozoal groups (1.08%) or the total set (1.09%). Closer examination of inter-domain associations showed the presence of larger microbial clusters. One comprised SK2, Clostridiales un2, *Clostridium*, *Metadinium minorum*, Clostridiales un1 and Bacteria1 and the second comprised *Clostridium* un2, *Succiniclasticum*, *Eudiplodinium*, *Neocallimastix*1 and Clostridiaceae un1. It seems that members of the Clostridiales order were very important in microbial networks while shifting from L to H diet. Examination of within-domain co-occurrences among fungi indicated that *Piromyces*3 and *Piromyces*6 as well as *Cyllamyces*1 and *Cyllamices*2 were positively correlated. No strong links between ciliate protozoa were observed. Among archaea, *Mbb*. *ruminantium* and *Mbb*. *gottschalkii* were in negative association, while the *Methanomassiliicoccaceae* Group 10 and *Methanomassiliicoccaceae* Group 8 WGK1 were in positive correlation. There were many other robust interactions between microorganisms belonging to all four different domains.

In the L-LSO comparison, no major difference was observed in the density of networks within bacteria (1.35%), as compared to density within archaea-fungi-ciliate protozoa groups (1.48%) or the total set (1.02%). One inter-domain, highly-linked cluster consisted of *Orpinomyces*1b, Bacteroidales un2, *Diplodinium* and Prevotellaceae un2, while a second cluster involved Lachnospiraceae un1, Succivibrionaceae un2, Ruminococcus1 and *Caecomyces*1. Within archaea, a strong positive correlation was observed between *Methanomassiliicoccaceae* Group 10 and *Methanomassiliicoccaceae* Group 8 WGK1, between *Methanomassiliicoccaceae* Group 12 ISO4-H5 and *Methanomassiliicoccaceae* Group 3b, and negative correlation between *Mbb*. *gottschalkii* and *Mbb*. *ruminantium*. Among fungi, *Cyllamices*1 and *Cyllamices*2 were in positive correlation as well as AL8 and SK2. Fungi were connected mainly with bacteria. In this oil-related dietary shift, strong correlations between ciliate protozoa were also observed. *Dasytricha* and *Raabena bella* were positively correlated, while *Entodinium* was negatively correlated with *Isotricha* LDK-2011. Among four domain interactions, no robust correlations were observed between bacteria and archaea but active interaction was detected between bacteria-fungi and bacteria-ciliate protozoa.

In the H-HSO comparison, the density of connections within bacteria was higher (2.54%) as compared with the density of connections within archaea and ciliate protozoa (1.73%) or in the total set (1.81%). No larger inter-domain clusters comprising members of all three domains were observed. Looking at bacteria—archaea interactions, Bacteroidales un2, *Methanomassiliicoccaceae* Group 8 WGK1 and *Succiniclasticum*, as well as another group of *Mbb*. *gottschalkii*, *Mbb*. *ruminantium*, Clostridia un, *Mmc*. Group 10 former linked clusters. Among archaea, only *Mbb*. *gottschalkii* and *Mbb*. *ruminantium* were negatively associated but there were many robust positive as well as negative correlations with bacteria. In contrast, among ciliate protozoa, positive correlations were observed between *Diplodinium*, *Ophryoscolex* and between *Isotricha* and *Metadinium minorum*, while a negative correlation occurred between *Entodinium* and *Isotricha* LDK 2011. However, no connections were observed between ciliate protozoa and archaea and only one was found between protozoa and bacteria.

## Discussion

Previous studies across a wide range of habitats have demonstrated that network based approaches can help in predicting the dynamics and structure of oceanic plankton ecosystems [11], reveal relations within microbial communities in soil [12] or highlight differences in taxa interactions within the human gut in health and disease [13]. Despite this development in other ecosystems, the rumen microbial community has not yet been widely explored by these methodologies. In this study we investigated advantages of stepwise increases in the depth of rumen microbial community analysis by evaluating microbial responses in a dietary intervention experiment. We applied standard alpha and beta diversity measurements and went a step further by introducing inter-domain co-occurrence network analysis.

Rumen microbial community is shaped by complex relationships between bacteria, ciliate protozoa, fungi and archaea. Different diets provide different primary substrates for fermentation; animal physiological features affect temperature, pH or feed retention time–factors that may cause the same microbial taxa to establish different symbiotic or antagonistic microbial interactions. Studying these interactions is difficult, as the ecological role of most of the ruminal taxa remains unknown and is impossible to replicate *in vitro*. Due to this complexity most culture based or molecular genetic studies have focused on one or two rumen microbial groups, without the ability to explore the ruminal microbiome as a whole. Recent transformations in the speed and cost of sequencing open new opportunities. Kittelmann et al. [9] for the first time presented simultaneously the whole rumen microbial community structure in three ruminant species and demonstrated non-random associations between some microbes at within- and inter-domain level. Kumar et al. [10] demonstrated diet and age effects on fungal, bacterial and archaeal taxa co-occurrence in dairy cows, suggesting that biotic and abiotic factors affecting rumen microbial community function still need to be better understood.

In this study we elucidated the effects of FC ratio and SO supplement on the ruminal bacteria, archaea, ciliate protozoa and fungi communities in dairy cows simultaneously. Plant oils are an attractive possibility to be used as feed additives to mitigate methane emissions in livestock. Both FC ratio and oil supplements have been demonstrated to alter protozoa [37,38], change methanogen diversity [39] and affect bacterial [1,40–43], or fungal [44] community structure, but the influence of lipid on the entire rumen microbial community is still largely unknown. Changes in one or several microbial species may be balanced by adjustments within the remaining rumen consortium, affecting not only diversity but also the function of the community. Understanding how lipid supplements modify the whole rumen microbial consortium is therefore crucial to understand better its effect on rumen fermentation and host metabolism in general.

### Rumen microbiome is shaped by diet composition

Inclusion of SO in the diet had no significant effect on total numbers of rumen bacterial, archaeal or protozoal communities but strongly affected fungi, as indicated by qPCR analysis. Although significant decreases in fungi were detected in both SO containing diets, the effect was more pronounced in HSO. Why forage rich diets supplemented with SO have less negative effect on fungi is not clear but it may be related to higher fungal diversity and particular fungal species being less sensitive to oil inhibition or indirectly to changes in general microbial metabolism within the rumen. In this study protozoa were not significantly decreased with the supplement of SO, even though toxicity of polyunsaturated FA to protozoa has been observed in sheep [37]. No significant decrease in the amount of protozoa was detected in response to various oil supplements in Nordic Red cattle diet [23,45], indicating that the composition of the basal diet and dosage of oil supplement may be among factors influencing oil effects on rumen protozoa.

The composition and diversity of ruminal bacteria were significantly influenced by diet, with higher proportions of Firmicutes detected in L diets and increased abundance of Bacteroidetes and Proteobacteria in H diets, as observed previously [22,46]. The abundance of known cellulolytic bacteria [47] was low in all diets. *Fibrobacter* was observed below 1%, while the *Ruminococcus* genus was not affected by dietary changes and was detected at 4% on average abundance in all diets. Although results are consistent with published studies [48–50], low abundance of the key cellulolytic bacteria may mean that other novel cellulolytic species may reside in rumen. Increases in abundance in the L diet of as yet uncharacterized groups of bacteria within *Clostridiaceae* and *Lachnospiraceae* families could have cellulolytic properties, supporting similar observations in gut of humans on higher fiber diets [51] or in global rumen census data on forage-rich diets [2]. In general, out of 23 diet-affected bacterial groups, 15 are still not identified at genus level. Among them were bacterial groups within *Prevotellaceae*, *Clostridiaceae*, *Lachnospiraceae*, *Ruminococcaceae* and *Succinivibrionaceae* families. These groups are relatively abundant, suggesting that their (unknown) metabolic role in the rumen ecosystem may be important. Further research in identifying unknown strains of microorganisms and clarifying their functional role is needed.

Methanogenic archaea indirectly stabilize the fermentation process in the rumen by utilizing H_2_ and reducing H_2_ partial pressure. In this study, the ruminal methanogenic community was dominated by *Mbb*. *ruminantium* and *Mbb*. *gottschalkii*. Both are H_2_ utilizers but their abundance correlated negatively with each other. Animals receiving forage diets often produce more methane, suggesting higher availability of H_2_ in the rumen for archaeal utilization. The higher proportion of *Mbb*. *gottschalkii* observed in the L treatment could indicate that it thrives better with other H_2_-producing microorganisms. *Methanosphaera*, usually a minor group of archaea in rumen [4], was found with all four dietary treatments at low abundance and was not susceptible to SO.

Ruminal protozoa contribute to fiber digestion, help control the rate of carbohydrate fermentation and may impact negatively on protein metabolism [52]. Higher protozoal diversity in L treatment may be beneficial for fiber utilization as up to 40% of fiber degradation in rumen may be associated with protozoal activity [53]. Addition of SO to the diet did not significantly decrease protozoal counts but negatively affected protozoal diversity, with a stronger effect in H compared to L diets. Our results confirm previous observations that *Entodinium* spp are less sensitive to SO or other lipids [37,54] as they dominated in all, but in particular in SO-containing diets.

Fungi initiate mechanical and enzymatic plant fiber degradation and enable access for secondary metabolizers. The higher diversity of anaerobic fungi in L diets observed in this as well as other studies [44,55] suggest that fungi are important for feed utilization efficiency and play an active role in shaping rumen conditions for bacteria, archaea and ciliates. Among well characterized genera, the predominant fungus in the L treatment was *Caecomyces*1. Its higher abundance may be related to complementary fibrolytic activity with *Fibrobacter*, as shown under *in vitro* conditions [56]. Addition of SO to the H diet suppressed overall fungal diversity but oil in the L diet increased the abundance of novel groups AL8, SK3 and SK2, while it decreased KF1 and MN3, and *Cyllamices*. Information on the functional properties of novel fungal groups is still missing. The fact that many of them were detected in our dataset indicates that novel groups actively share a specific niche within the microbial community and react strongly to changing physico-chemical conditions within the rumen.

#### Rumen microbial taxa form diet-specific networks

Microbial interaction patterns are best understood when samples are collected from environments undergoing shifts in specific characteristics that cause sufficient changes in microbial abundance. Here we evaluated networks of rumen microbial taxa responding to the changing proportions of fiber and concentrates (L-H); and microbial responses to SO in diets containing high (H-HSO) or low (L-LSO) proportion of concentrates. We created undirected networks by calculating pairwise partial correlations instead of correlations for each pair of taxa. While a correlation network would imply interactions between taxa even when the connection is indirect, for example when both are controlled by a third taxon, partial correlation network does not include such indirect interactions in the network. The second challenge in constructing robust associations between microbial taxa is the small sample size normal in animal experimental trials. Several approaches and statistical models have been created and tested [57], showing that specific benefits related to a particular method are visible in big data sets (n > 1000), while their performance in small sample sets is similar. The ‘graphical lasso’ performs reasonably well in small data sets and therefore was used in this analysis.

We explored within- and between-domain microbial associations and showed that they are diet-specific. This reflects that the diets chosen for comparison contained different types of fermentable substrates, thereby defining biochemical processes leading to specific microbial interactions. We explored the role of bacteria, archaea, ciliate protozoa and fungi in the organization of association networks. Although bacteria is the most diverse group of microorganisms in the rumen, we did not find evidence for bacteria to play a more central or peripheral role in the rumen biological networks as compared to microorganisms from other domains. However, bacteria formed stronger associations with each other as compared to interactions with ciliate protozoa, fungi or archaea when only the most robust correlations were evaluated. The graphical model of interaction between taxa nodes does not differentiate between direct nutritional and indirect specific niche sharing associations. Nevertheless, it indicates that there is substantial interaction between certain sets of bacteria in response to dietary changes.

Another novel general observation was that no strong associations were detected between archaea and ciliate protozoa in all three diet-change cases. Associations between protozoa and archaea are of high relevance because of the H_2_ transfer between them and their role in methane production. The lack of strong archaea-protozoa associations in this study supports similar observations reported by [2], suggesting less specific and more random associations between archaea and protozoa in their interactions in *in vivo* environments.

Our results suggest that bacteria-archaea interactions respond to SO but may be extremely diet sensitive. Addition of SO to concentrate rich diet (H-HSO) stimulated the strong positive and negative associations between bacteria and *Methanobrevibacter*, as well as *Methanomassiliicoccaceae*. More specifically, positive co-occurrences were observed between *Mbb*. RT and *Fibrobacter*, *Mbb*. *wolinii* and Clostridiales un1, *Mmc*. Group 10 and Clostriadia un2, *Mmc*. Group8 WGK1 and *Succiniclasticum*. Negative co-occurrence was observed between *Prevotella* and *Methanimicrococcus blatticola*, *Mbb*. *ruminantium* and Clostridia un2, *Mmc*. Group8 WGK1 and Bacteroidales un2. In contrast, when SO was added to the forage-rich diet (L-LSO), no strong correlation between bacteria and archaea was detected. In the global rumen census [2], positive associations were detected between less abundant rather than most dominant bacteria and archaea. Similarly, in this study many bacteria associated with archaea were low in abundance and not yet identified at genus level, therefore the nature of these inter-domain interactions remains to be explored.

Similarly, diet specificity was observed also in bacteria-ciliate protozoa associations. Most of the strongest positive as well as negative co-occurrences were detected either when diet composition shifted from high forage to concentrate (L-H) or when SO was added to the L but not the H diet. In the first case, *Metadinium minorum*, *Eudiplodinium* and *Isotricha* were co-occurring with some bacterial taxa, while in the second case a similar co-occurrence was found with *Metadinium minorum* and *Diplodinium*. Some positive associations between bacteria and ciliate protozoa, especially between *Isotricha* and *Dasytricha* with *Fibrobacter* were detected in the global rumen census [2]. Although no such association was observed here, differences between the studies may reflect variations in dietary background.

Rumen anaerobic fungi formed positive associations with each other and with microorganisms from other domains. In particular, many positive associations were observed between bacteria, mainly from the Firmicutes phylum and fungi in L-LSO comparison: *Caecomyces*1—*Ruminococcus*1, *Piromyces*3—*Succiniclasticum*, *Piromyces*7—Lachnospiraceae un2, MN3—Catabacteriaceae un. Considering that diets high in forage stimulate proliferation of fungi and Firmicutes, strong associations detected between some of them may represent more specific interactions or could be due to the availability of common metabolites.

We explored the organization of microbial co-occurrence networks by looking for the existence of main hub(s), as strong regulation of bacterial interactions was suggested in the human gut microbiome [58]. In this study, most of the microorganisms serving as main hubs in microbial networks were low abundance taxa. These observations are consistent with the human gut data, where very low abundance *Christensenellaceae* (Firmicutes order Clostridiales) was identified as highly heritable bacteria serving as a hub in gut bacterial networks [58]. In the rumen, not only bacteria but also some ciliate protozoa, fungi and archaea were identified as hubs, suggesting a complex involvement of all four domains in microbial interaction regulation. Interestingly, each specific diet change analysed had own set of taxa serving as central hubs, suggesting that, although a core microbiome can be identified across different diets and hosts [2], diet is the regulating factor defining which of the taxa act as hubs and govern microbial interactions.

The diet induced changes in rumen microbial community structure are related to changes in ruminal VFA synthesis or methane production. A common approach to explore which diet affected taxa could be related to changes in particular phenotype is by performing taxa abundance—phenotype regression analysis. Using standard diversity analyses it is however not possible to deduce whether microbial taxa significantly associated with animal phenotype work in isolation or are members of more complex networks. Therefore, we explored the behavior of the microbial taxa significantly affected by diet in co-occurrence networks and found that many of them co-occurred together in the more stringent correlation networks. These microbial clusters would be the first candidates to study *in vitro* and/or via metagenome and metatranscriptome approaches to understand biological interaction mechanisms leading to specific phenotypes.

## Conclusions

The various layers of community structure analysis currently available provide a wealth of information about the abundance of different microbial taxa and their response to dietary changes. Here, network analysis performed using ssu rRNA amplicon sequence data expanded on the information obtained from standard diversity methods. It predicted taxa at hubs of interaction, and interactions between taxa, that mostly as yet have no mechanistic explanation. Validation of these interactions will require basic investigation of the physiology of the microorganisms and should be complemented by metagenomics as well as metatranscriptomics analyses of microbiome genes and gene networks.

## Supporting information

S1 Text Supporting information, containing supporting methods, supporting data, and supporting references. (DOCX)

S1 Table Sequences of primers used for qPCR and metabarcoding amplicon sequencing. (DOCX)

S2 Table Rumen bacterial, archaeal, ciliate protozoa and fungal taxa average abundances per diet and Tukey test for pairwise comparisons (*P* values). (XLSX)

S1 Fig Bacterial, archaeal, protozoal and fungal richness as measured from amplicon sequence data, in four diets. Diets are as follows: high (H) or low (L) proportion of concentrates without oil, or supplemented with SO (HSO and LSO, respectively).
(TIF)

S2 Fig Bacterial, archaeal, protozoal and fungal evenness as measured from amplicon sequence data, in four diets. Diets are as follows: high (H) or low (L) proportion of concentrates without oil, or supplemented with SO (HSO and LSO, respectively).
(TIF)

S3 Fig Graphical network representing the interactions between rumen microorganisms within L-H diet comparison. Nodes correspond to microbial taxa while green and red edges represent positive and negative partial correlations above 0.05, respectively. Microbial taxa are colored by taxonomy: archaea—gray, ciliate protozoa—blue, fungi—dark yellow, bacteria—white.
(PDF)

S4 Fig Graphical network representing the interactions between rumen microorganisms within L-LSO diet comparison. Nodes correspond to microbial taxa while green and red edges represent positive and negative partial correlations above 0.05, respectively. Microbial taxa are colored by taxonomy: archaea—gray, ciliate protozoa—blue, fungi—dark yellow, bacteria—white.
(PDF)

S5 Fig Graphical network representing the interactions between rumen microorganisms within H-HSO diet comparison. Nodes correspond to microbial taxa while green and red edges represent positive and negative partial correlations above 0.05, respectively. Microbial taxa are colored by taxonomy: archaea—gray, ciliate protozoa—blue, bacteria—white.
(PDF)
