# Supplementary material for: Taxon abundance, diversity, co-occurrence and network analysis of the ruminal microbiota in response to dietary changes in dairy cows
Source: PLoS One. 2017 Jul 13;12(7):e0180260. doi: 10.1371/journal.pone.0180260 (PMC5509137; doi:10.1371/journal.pone.0180260)
Supplement: S1 Text — (DOCX) [file pone.0180260.s001.docx]

**Supporting methods**

*Amplicon preparation and sequencing on GS Junior (Roche).*

The PCR was performed in a total volume of 20 µl containing 50 ng of template DNA, 0.5µM of each primer (without barcode), 200µM each dNTP, 2mM MgCl_2_, 1 x Phusion HF Buffer, and 0.4 U of Phusion HF DNA Polymerase (Thermo Scientific). Amplification was carried out using 2 step protocol with initial denaturation at 98°C for 30s, followed by 35 cycles of 98°C for 10s and 72°C for 10s, and final extension at 72°C for 5 min. Negative control containing miliq H_2_O instead of template DNA was included in parallel. PCR products were visualized on 1 % agarose gel and cleaned using MinElute Reaction Cleanup Kit (QIAGEN). A second PCR was performed under the same conditions by using barcoded primers and 1 µl of cleaned amplicon as a template. Each sample was done in triplicate, PCR products were pooled, run on 1% agarose gel and 450bp fragment was cut and purified from the gel using GFX PCR DNA and Gel Band purification kit (GE Healthcare). Samples were eluted in 40µl of Tris-HCl and amplicon concentration was measured using Qubit dsDNA HS Assay Kit (life technologies). Two amplicon pools were created with 5 samples each by pooling them in equimolar concentrations. Five hundred ng of each pool was purified with MinElute Reaction Cleanup Kit (QIAGEN) and eluted in 16ul TE buffer. Amplicon sequencing was performed using GS Junior (Roche) at former MTT Agrifood Research Finland following the standard procedures.

**Supporting data**

*Depth of metabarcoding sequencing*

Sequencing of sixteen samples resulted in 101,888 non-chimeric high quality reads of bacteria, 3,296,653 of archaea, 1,338,897 of ciliate protozoa and 164,759 reads of fungi, respectively. From HSO treatment one sample failed repeated attempts to sequence protozoa and three samples failed sequencing of fungal communities and were excluded. To obtain unbiased diversity estimates, a sample-based rarefaction analysis was performed using the sequencing depth of the sample with fewest reads: 3700 for bacteria, 10,000 for archaea, 40,000 for ciliate protozoa and 3000 for fungi.

**Supporting references**

59. Maeda H, Fujimoto C, Haruki Y, Maeda T, Kokeguchi S, Petelin M, et al. Quantitative real-time PCR using TaqMan and SYBR Green for Actinobacillus actinomycetemcomitans, Porphyromonas gingivalis, Prevotella intermedia, tetQ gene and total bacteria. FEMS Immunology & Medical Microbiology. 2003 Oct 1;39(1):81-6.

60. Sylvester JT, Karnati SK, Yu Z, Morrison M, Firkins JL. Development of an assay to quantify rumen ciliate protozoal biomass in cows using real-time PCR. The Journal of Nutrition. 2004 Dec 1;134(12):3378-84.

61. Denman SE, Tomkins NW, McSweeney CS. Quantitation and diversity analysis of ruminal methanogenic populations in response to the antimethanogenic compound bromochloromethane. FEMS Microbiology Ecology. 2007 Dec 1;62(3):313-22.

62. Denman SE, McSweeney CS. Development of a real-time PCR assay for monitoring anaerobic fungal and cellulolytic bacterial populations within the rumen. FEMS Microbiology Ecology. 2006 Dec 1;58(3):572-82.

63. Tuckwell DS, Nicholson MJ, McSweeney CS, Theodorou MK, Brookman JL. The rapid assignment of ruminal fungi to presumptive genera using ITS1 and ITS2 RNA secondary structures to produce group-specific fingerprints. Microbiology. 2005 May 1;151(5):1557-67.

64. Jami E, Mizrahi I. Composition and similarity of bovine rumen microbiota across individual animals. PloS ONE. 2012; 7(3):e33306.
