# Supplementary material for: Taxon abundance, diversity, co-occurrence and network analysis of the ruminal microbiota in response to dietary changes in dairy cows
Source: PLoS One. 2017 Jul 13;12(7):e0180260. doi: 10.1371/journal.pone.0180260 (PMC5509137; doi:10.1371/journal.pone.0180260)
Supplement: S1 Table — (DOCX) [file pone.0180260.s002.docx]

**S1 Table.** **Sequences of primers used for qPCR and metabarcoding amplicon sequencing**.

| **Target group** | **Primer name** | **Nucleotide sequence** | **T (ᵒC)** | **Amplicon size (bp)** | **Reference** |
| --- | --- | --- | --- | --- | --- |
| **qPCR** |  |  |  |  |  |
| All bacteria | UniF | GTGSTGCAYGGYTGTCGTCA | 61 | 120 | [59] |
|  | UniR | ACGTCRTCCMCACCTTCCTC |  | 16S rRNA |  |
|  |  |  |  |  |  |
| All protozoa | 316f | GCTTTCGWTGGTAGTGTATT | 55 | 223 | [60] |
|  | 539r | CTTGCCCTCYAATCGTWCT |  | 18S rRNA |  |
|  |  |  |  |  |  |
| Methanogens | F | TTCGGTGGATCDCARAGRGC | 56 | 140 | [61] |
|  | R | GBARGTCGWAWCCGTAGAATCC |  | *mcr*A gene |  |
|  |  |  |  |  |  |
| All fungi | F | GAGGAAGTAAAAGTCGTAACAAGGTTTC | 62 | 120 | [62] |
|  | R | CAAATTCACAAAGGGTAGGATGATT |  | between 18S rRNA and ITS1 |  |
| **Amplicon sequencing** |  |  | **Target region** | |  |
|  |  |  |  | |  |
| Archaea | ArchF | CCTGCTCCTTGCACACAC | 16S rRNA V9 | | [23] |
|  | ArchR | CCTACGGCTACCTTGTTAC |  | |  |
| Bacteria | 107F | GGCGVACGGGTGAGTAA | 16S rRNA V2-3 | | [64] |
|  | 530R | CCGCNGCNGCTGGCAC |  | |  |
| Protozoa | 316f | GCTTTCGWTGGTAGTGTATT | 18S rRNA | | [60] |
|  | 539r | CTTGCCCTCYAATCGTWCT |  | |  |
| Fungi | MN100F | TCCTACCCTTTGTGAATTTG | ITS1 | | [63] |
|  | MNGM2 | CTGCGTTCTTCATCGTTGCG |  | |  |
