# Supplementary figures and images for: Taxon abundance, diversity, co-occurrence and network analysis of the ruminal microbiota in response to dietary changes in dairy cows
Source: PLoS One. 2017 Jul 13;12(7):e0180260. doi: 10.1371/journal.pone.0180260 (PMC5509137; doi:10.1371/journal.pone.0180260)

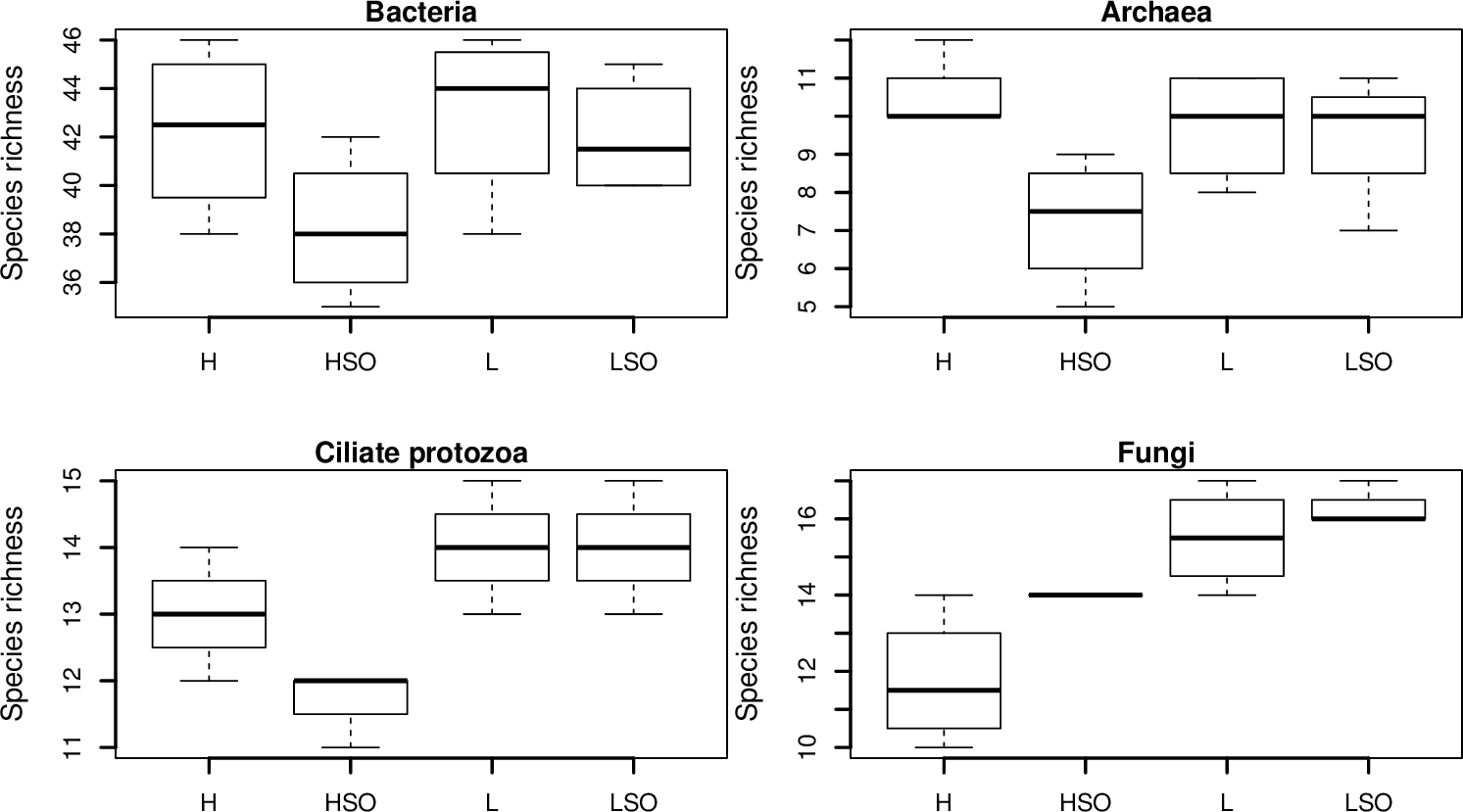

Supplement: S1 Fig — Diets are as follows: high (H) or low (L) proportion of concentrates without oil, or supplemented with SO (HSO and LSO, respectively). (TIF) [file pone.0180260.s004.tif]

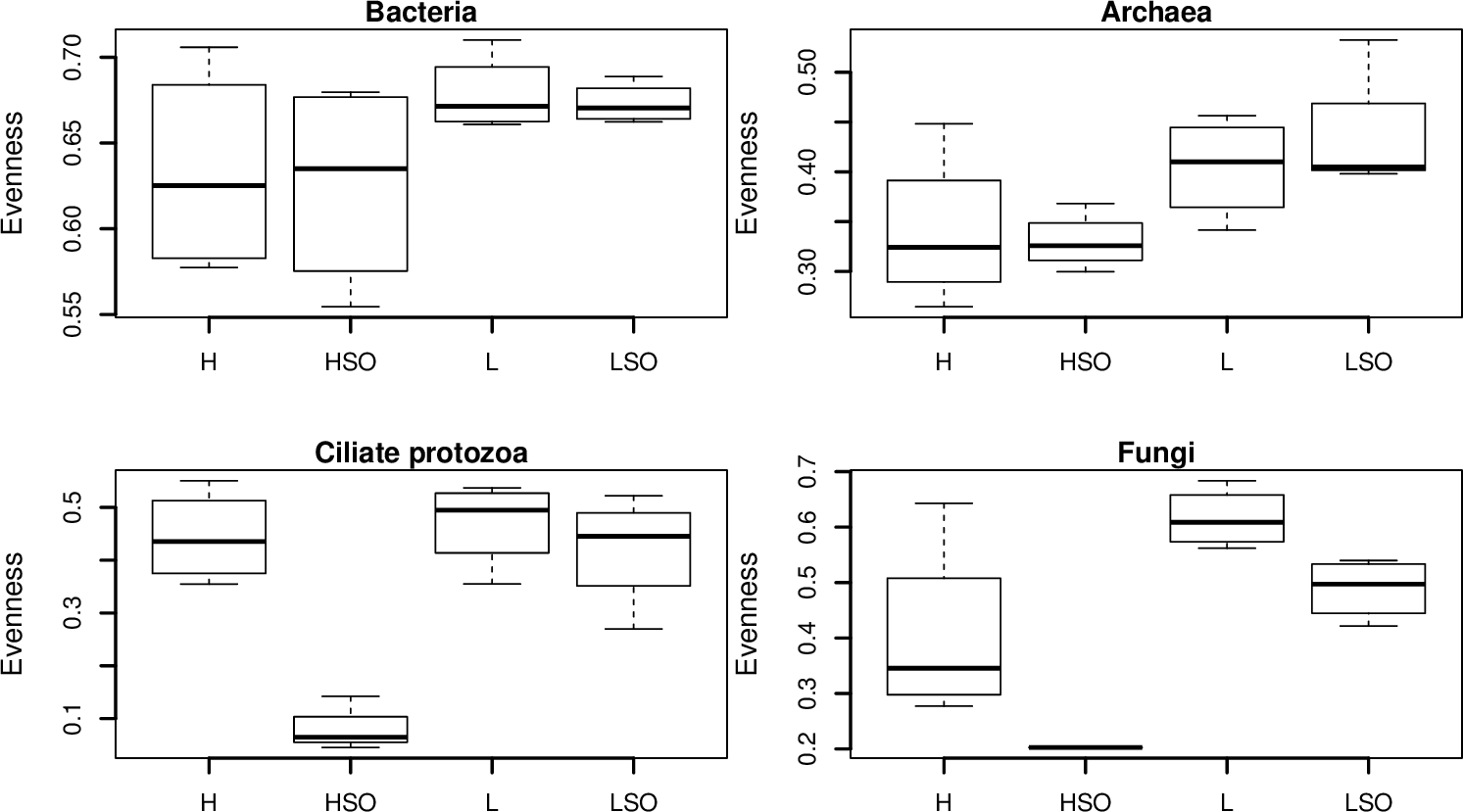

Supplement: S2 Fig — Diets are as follows: high (H) or low (L) proportion of concentrates without oil, or supplemented with SO (HSO and LSO, respectively). (TIF) [file pone.0180260.s005.tif]

**L-H, partial correlation>0.05**

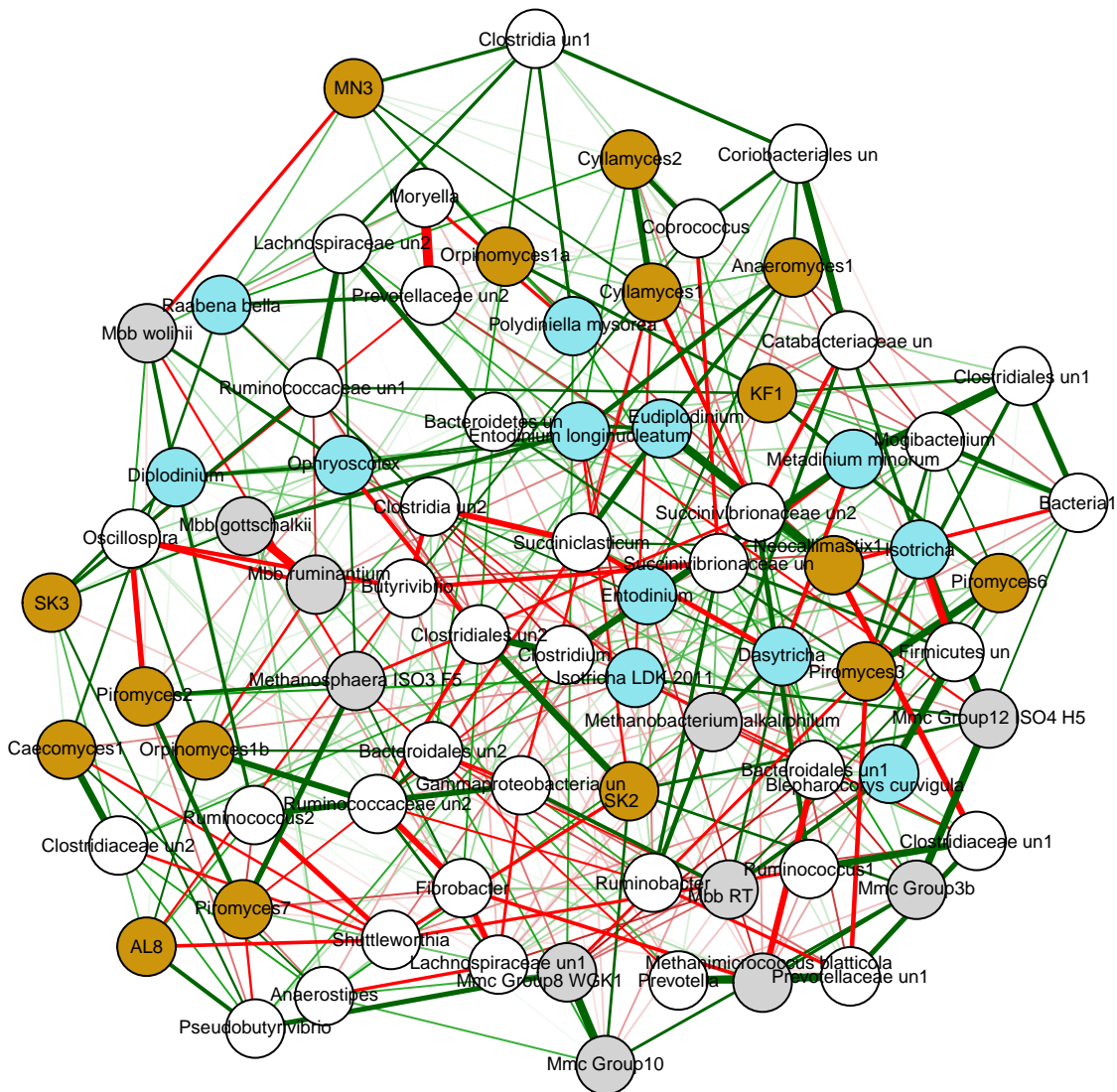

Supplement: S3 Fig — Nodes correspond to microbial taxa while green and red edges represent positive and negative partial correlations above 0.05, respectively. Microbial taxa are colored by taxonomy: archaea—gray, ciliate protozoa—blue, fungi—dark yellow, bacteria—white. (PDF) [file pone.0180260.s006.pdf]

# L-LSO, partial correlation>0.05

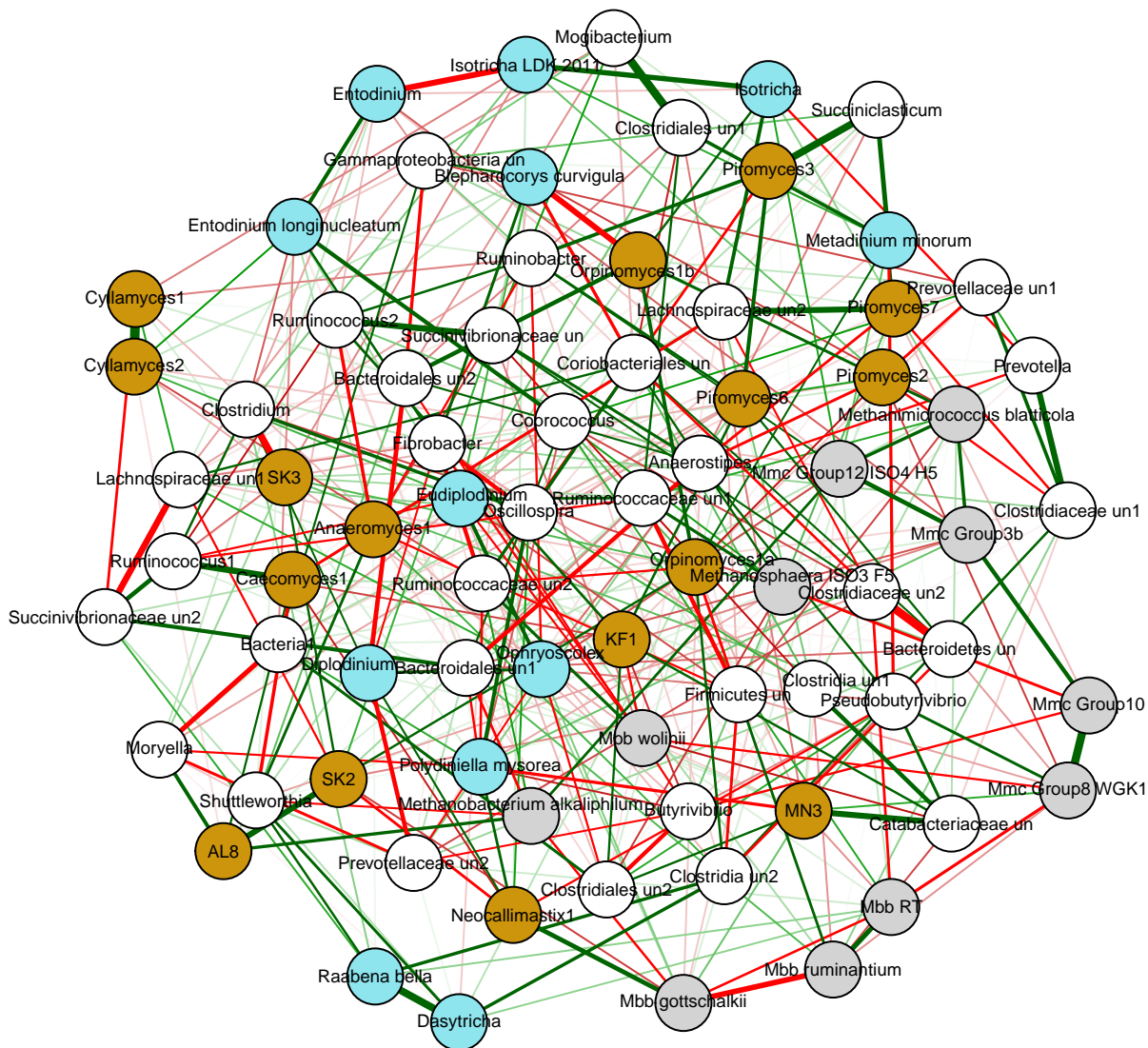

Supplement: S4 Fig — Nodes correspond to microbial taxa while green and red edges represent positive and negative partial correlations above 0.05, respectively. Microbial taxa are colored by taxonomy: archaea—gray, ciliate protozoa—blue, fungi—dark yellow, bacteria—white. (PDF) [file pone.0180260.s007.pdf]

**No fungi, H-HSO, partial correlation>0.05**

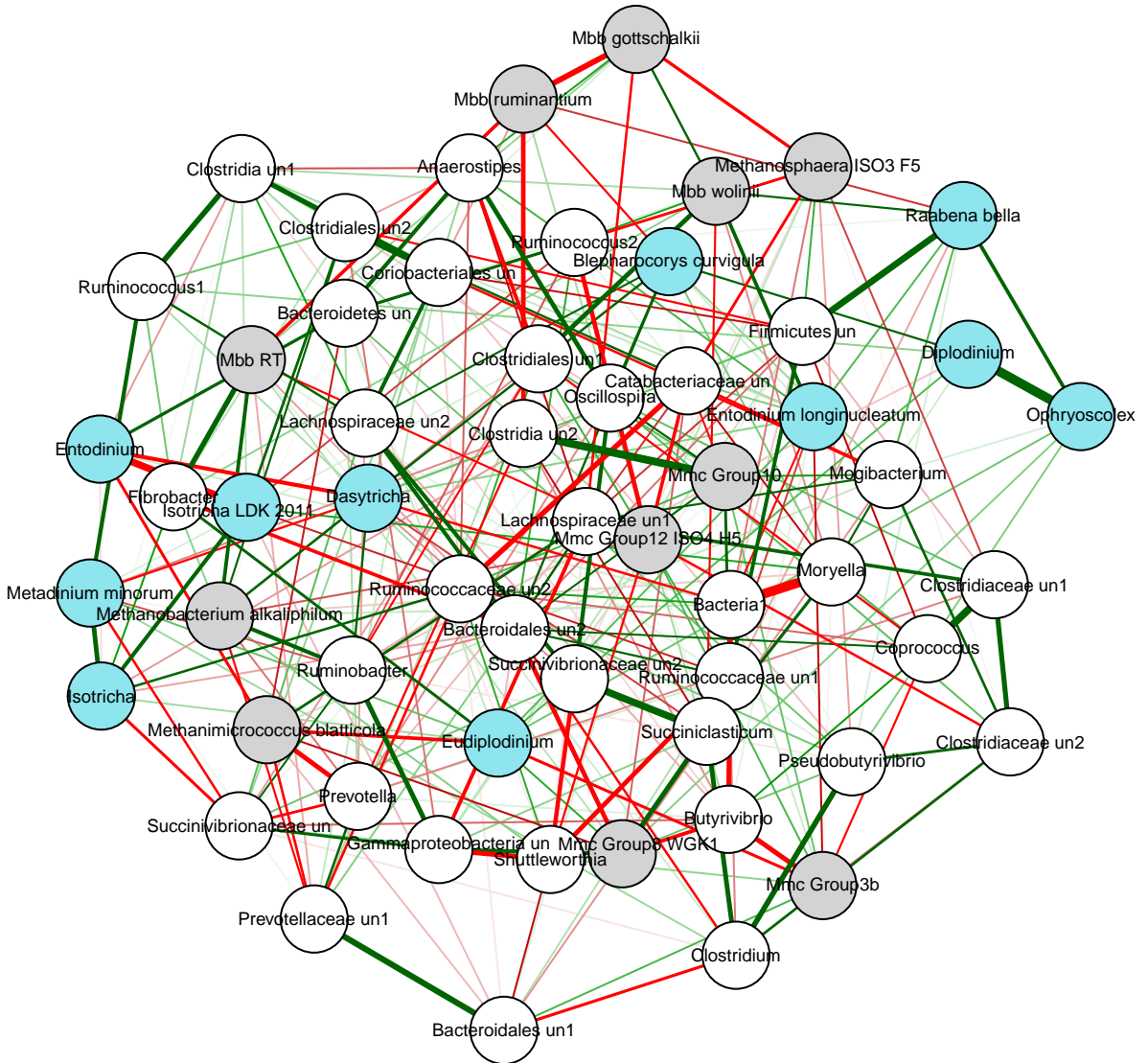

Supplement: S5 Fig — Nodes correspond to microbial taxa while green and red edges represent positive and negative partial correlations above 0.05, respectively. Microbial taxa are colored by taxonomy: archaea—gray, ciliate protozoa—blue, bacteria—white. (PDF) [file pone.0180260.s008.pdf]
